# Effect of Sperm Cryopreservation on miRNA Expression and Early Embryonic Development

**DOI:** 10.3389/fcell.2021.749486

**Published:** 2021-12-22

**Authors:** Xiaoyu Xu, Wanqiong Li, Lina Zhang, Yazhong Ji, Jiaying Qin, Lu Wang, Mingwen Wang, Lingbin Qi, Jinfeng Xue, Bo Lv, Xunyi Zhang, Zhigang Xue

**Affiliations:** ^1^ Department of Regenerative Medicine, Tongji University School of Medicine, Shanghai, China; ^2^ Reproductive Medicine Center, Tongji Hospital, Tongji University School of Medicine, Shanghai, China

**Keywords:** gamete cryopreservation, sperm cryopreservation, miRNA expression, embryonic development, miR-148b-3p

## Abstract

Although sperm preservation is a common means of personal fertility preservation, its effects on embryonic development potential need further investigation. The purpose of this study was to identify key microRNA (miRNA) in cryopreserved sperm and determine the changes of these miRNAs and their target genes during embryonic development using cryopreserved sperm. Moreover, the embryonic development potential of cryopreserved sperm was estimated in assisted reproductive technology (ART), where key miRNAs and target genes were validated in sperm and subsequent embryos. Clinical data of embryonic development from cryopreserved sperm indicated a significant decrease in fertilization rate in both *in vitro* fertilization (IVF) and intracytoplasmic sperm injection (ICSI) cases, as well as a reduction in blastocyst formation rate in ICSI cases. Meanwhile there was a significant increase in blocked embryo ratio of Day1, Day2, and Day3.5 embryos when frozen-thawed mouse sperm was used, compared with fresh mouse sperm, suggesting a potential negative effect of sperm cryopreservation on embryonic development. From frozen-thawed and fresh sperm in humans and mice, respectively, 21 and 95 differentially expressed miRNAs (DEmiRs) were detected. miR-148b-3p were downregulated in both human and mouse frozen-thawed sperm and were also decreased in embryos after fertilization using cryopreserved sperm. Target genes of miR-148b-3p, Pten, was identified in mouse embryos using quantitative real-time PCR (qRT-PCR) and Western blot (WB). In addition, common characters of cryopreservation of mouse oocytes compared with sperm were also detected; downregulation of miR-148b-3p was also confirmed in cryopreserved oocytes. In summary, our study suggested that cryopreservation of sperm could change the expression of miRNAs, especially the miR-148b-3p across humans and mice, and may further affect fertilization and embryo development by increasing the expression of Pten. Moreover, downregulation of miR-148b-3p induced by cryopreservation was conserved in mouse gametes.

## Introduction

With the ever-increasing worldwide rates of cancer, the postponement of childbearing, and the impact of environment and epidemics on reproductive health, fertility preservation—which includes the frozen storage of sperm, oocytes, embryos, and reproductive tissues—is a meaningful option to retain fertility before damage occurs, such as chemotherapy, radiation, infection, or the decline of sperm quality with increasing age (Zambrano et al., 2020; Oktay et al., 2021). Cryopreservation of sperm is a well-used technique in reproductive medicine, but it may be harmful to sperm (Henry et al., 1993; Gandini et al., 2006; Nangia et al., 2013). A complex change including ice crystal formation, osmotic changes, and physical and chemical stress during the cryopreservation process contribute to the damage to the cell membrane, internal structures, or gene expression, especially using the rapid or slow freezing techniques (Hezavehei et al., 2018). Although vitrification reduces these effects and improves the survival rate, the cryopreservation of sperm, no matter which freezing technology, could still lead to significant changes in motility, DNA integrity, reactive oxygen species (ROS) levels, fertilizing ability, and gene expression profiles induced by these physical, chemical, and biological factors (Hezavehei et al., 2018; Valipour et al., 2021). Therefore, further investigation of this subject will be beneficial for the development of sperm cryopreservation technologies and will assist people with fertility preservation, whether for medical or social needs.

Mature spermatozoa are generally transcriptionally and translationally inert cells. Nearly all sperm RNAs are microRNA (miRNA); other RNA components include low levels of mRNAs, Piwi-interacting RNA (piRNA), and repeat-associated small RNAs (Henry et al., 1993; Hamatani, 2012). miRNAs, a class of 22 nt and single-stranded RNA molecules, have functions in post-transcriptional regulation of gene expression and mediate epigenetic inheritance (Abu-Halima et al., 2020). Most miRNAs are highly conserved across species due to their critical biological functions (Pantano et al., 2015). Increasing evidence found that many miRNAs affected by the sperm cryopreservation could associate with early embryonic development in mammals, such as miR-34c, which is decreased in sperm after being frozen-thawed and further affects the development of preimplantation embryos in humans and mice (Lee et al., 2009; Liu et al., 2012; Shi S. et al., 2020); miR-26a plays critical roles in embryo formation (Abu-Halima et al., 2020), while miR-19b-3p is a potential biomarker to predict pregnancy outcome in humans (Krawczynski et al., 2015). In addition, paternal miRNAs can contribute to the degradation of maternal mRNAs, the activation of zygotic genes, and establishment of the key pluripotency transcriptional genes, indicating that miRNAs play a critical role in developmental processes (Abu-Halima et al., 2020).

The objective of the current study was to explore the potential mechanism and effect of sperm cryopreservation in subsequent early embryo development. We evaluated the embryonic development of frozen-thawed sperm from clinical data and mouse experiments. Then, using miRNA sequencing, we identified the key conserved differentially expressed miRNAs (DEmiRs) between frozen-thawed and fresh sperm. Next, using quantitative real-time PCR (qRT-PCR), we identified key miRNAs and their target genes in embryos that developed following sperm cryopreservation. In addition, we analyzed common effects on murine gametes, both sperm and oocytes. Our data showed that differentially expressed miR-148b-3p in sperm might be associated with fertilization and embryo formation.

## Manuscript Formatting

### Materials and Methods

#### Ethics Statement

The animal experimental procedures were approved by the Institutional Animal Care and Use Committee of Tongji University and the Ethics Committee of the Institute of Animal Science, Tongji University (TJAA06420101). All human sperm samples were donated for research by patients who had provided informed consent at Tongji Hospital, with the approval of the Institutional Ethical Committee for Scientific Research (2019-062).

#### Retrospective Cohort Study

The retrospective study was conducted at Tongji University Hospital. Research protocols adhered to relevant guidelines and regulations completely. The study of sperm cryopreservation included female patients aged 25–30 years who underwent treatments of *in vitro* fertilization (IVF) or intracytoplasmic sperm injection (ICSI) with an indication of tubal abnormality, sequelae of pelvic inflammatory disease, male factor, or unexplained infertility from January 2016 to September 2020. The anti-Müllerian hormone (AMH), antral follicle count (AFC), basal follicle-stimulating hormone (FSH), and luteinizing hormone (LH) levels in eligible women were also normal. Male sperm indicators were evaluated according to the criteria of the 5th WHO laboratory manual (Cao et al., 2011). Semen with normal parameters, as defined by 1) total sperm count ≥39 * 10^6^; 2) sperm concentration ≥15 * 10^6^/ml; 3) PR (progressive motility) +NP (non-progressive motility) ≥40%, PR ≥ 32%; 4) sperm normal morphology ≥4%, DNA fragmentation index (DFI) < 15%; and 5) normal infectious index, were selected. In addition, informed consent was not needed, because of the retrospective nature of the study.

#### Human and Mouse Sperm Cryopreservation and Thawing

Mouse sperm were cryopreserved using R18S3 (Easycheck, Nanjing, China). In brief, adult male mice were sacrificed by cervical dislocation. Cauda epididymides were carefully dissected by separating fat, blood vessels, and ligaments and placed into a 120-µl drop of cryoprotectant (CPA) (Easycheck, Nanjing, China) to remove any blood and fat globules. The tissue was then transferred into a new CPA drop, where a 1-ml syringe needle was used to carefully pierce the cauda epididymides and to agitate the tissues gently to liberate the sperm. Subsequently, the sperm were moved from the dish into cryogenic vials (Corning, NY, United States), were placed in liquid nitrogen (LN) vapor (3–5 cm above the liquid level) for 10–15 min, followed by plunging into LN, and then stored for at least 1 week. Samples were thawed by incubation at 37°C for 3 min in a water bath with gentle agitation then moved into an incubator (37°C and 5% CO_2_) for 45 min (Khanlari et al., 2021).

Sperm Freezing Medium (Origio, Malov, Denmark) was used for human semen cryopreservation as described previously (Henry et al., 1993). Firstly, each semen sample was diluted (1:1) with freezing medium (Origio). After 10 min at 37°C for equilibration, the mixture was placed into cryogenic vials (Corning). The tubes were then frozen in LN vapor for 30 min and plunged into LN for storage (Abush et al., 2014). Human sperm samples were thawed through incubation at 37°C for 3 min in a water bath with gentle agitation.

#### Mouse Oocyte Cryopreservation and Thawing

Oocytes were vitrified using Vit Kit-Freeze (Easycheck, Nanjing, China). Briefly, almost 260 oocytes (from 10 ICR mice) were exposed to equilibration solution (ES) medium for 4 min, transferred to vitrification solution (VS) for 1 min, then loaded on a cryotop device (Easycheck, Nanjing, China) within 30 s, and finally plunged into LN (Yang et al., 2016).

All oocytes were thawed by Vit Kit-Thaw (Easycheck, Nanjing, China), which include four bottles of thawing solution: DM-1, DM-2, DM-3, and WM. In short, oocytes were placed in 200 µl thawing solution (DM-1) at 37°C for 1 min and transferred to two drops of thawing solution (DM-2 and DM-3) in turn, each for 3 min, then were put into another thawing solution (WM) for 3 min and cultured later in incubators (5% CO_2_ and 37°C) for 30 min to recover (Barberet et al., 2020).

### *In Vitro* Fertilization

For mouse superovulation, female mice were injected with 5–10 IU of pregnant mare serum gonadotropin (PMSG), followed by an injection of 5–10 IU human chorionic gonadotropin (hCG) 48 h later. After a further 14–16 h, the cumulus–oocyte complexes (COCs) were collected from the mouse oviducts (Yao et al., 2019).

For mouse IVF, cumulus–oocyte complexes were placed in a droplet of frozen-thawed or fresh sperm capacitated in HTF for 6–8 h in the incubator (37°C, 5% CO_2_). Subsequently, zygotes were quickly washed three times in KOSM media and then cultured in KSOM. The mouse embryo culture continued till Day3.5 in an incubator set at 37°C and 5% CO_2_ (Dumollard et al., 2008; Karimi et al., 2017; Azizi et al., 2020).

#### RNA Extraction, Small RNA Library Construction, Sequencing, and Data Pre-Processing

In our experiment, three kinds of samples, human semen, mouse sperm, and mouse oocyte, were used for miRNA sequencing. Considering the low miRNA content in sperm and oocytes, we collected multi-samples directly for RNA extraction in order to meet the requirements for miRNA sequencing, then the RNA was used to construct the cDNA library; the same amount of cDNA samples was input for miRNA sequencing.

Each human sperm sample consisted of a combination of three human semen samples in a group, while each mouse sperm sample was a mixture of five murine semen samples, and each mouse oocyte sample consisted of 200–300 oocytes from 10 female mice. Each fresh group and frozen-thawed group included three mixed samples. Then total RNA was extracted using Trizol reagent (Invitrogen, CA, United States). The quality and integrity of RNA were evaluated using an RNA 6000 Nano LabChip Kit (Agilent, CA, United States) (Yuan et al., 2015). The miRNA libraries for samples were constructed and sequenced using the Illumina Hiseq-2000 platform. Concisely, each sample of genome RNA (gRNA) was used to construct miRNA libraries using TruSeq Small RNA Sample Prep Kits (Illumina, CA, United States). The miRNA was ligated with 3′- and 5′- end adapters, then first-strand cDNA was synthesized and subjected to PCR amplification. Afterwards, the DNA was purified by electrophoresis on a 6% Novex TBE page gel at 145 V for 60 min. The amplified DNA fragments (18–26 nt) were used to construct miRNA libraries, while library quality was assessed using a High Sensitivity DNA Chip Kit (Agilent, CA, United States) (Tsaur et al., 2007). The raw data were processed by using ACGT101-miR (LC Sciences, TX, United States). The 3′ adaptor and junk reads were removed; subsequently, high-quality reads with a length of 18–26 nt were selected. The clean data were aligned with miRBase 22.0 (human and mouse) by BLAST search (McDonald et al., 2011; Kozomara and Griffiths-Jones, 2014; Solly et al., 2017).

### Identification of Differentially Expressed miRNAs

Based on normalized deep-sequencing counts, the DEmiRs of oocyte samples were selected by using Fisher's exact test and chi-squared 2 × 2 test; a *p*-value of <0.05 was considered as the threshold.

DEmiRs of sperm samples, both human and mouse, were identified using analysis of variance (ANOVA) for multiple comparisons. The significance threshold was set to 0.05 in each test. In addition, most novel and conserved miRNAs were lowly expressed; thus, these miRNAs were excluded (Rybak et al., 2009; Xiong et al., 2014).

#### The Prediction of Target Genes and Functional Enrichment Analysis

MiRwalk3.0 (https://mirwalk.umm.uni-heidelberg.de), which includes three miRNA-target prediction programs (miRDB, miRTarBase, and Targetscan), was used to predict the target genes of DEmiRs. Overlapped target genes with at least two prediction programs were considered as the potential target genes. Functional enrichment analysis of the target genes was performed using miRwalk 3.0 and DAVIAD (https://david.ncifcrf.gov/); only required categories with an adjusted value of *p* < 0.05 were considered significantly enriched (Mazzeo et al., 2018).

### qRT-PCR for Key miRNAs and Target Genes

We set out to evaluate the expression of key miRNAs and target genes using qRT-PCR. Firstly, total sample RNA, including human sperm samples, mouse sperm, mouse oocytes, and 120–170 embryos, were isolated, then the first-stranded cDNA was synthesized using a PrimeScript ™ RT reagent kit (Takara Bio, Kusatsu, Japan); the reverse transcription reaction was performed at 37°C for 15 min, 42°C for 30 min, and 85°C for 5 s; qRT-PCR was performed with a TB Green Premix Ex Taq™ II kit (Takara Bio, CA, United States) on a Realtime PCR instrument (Roche); the PCR amplification conditions were 85°C for 10 s, followed by 40 cycles of 95°C for 5 s, and 60°C for 30 s (Alexandri et al., 2020).

In our study, all qRT-PCR experiments were repeated a minimum of three times; U6 (miRNA) and β-actin (target gene) were used as the reference gene. The relative expression level of miRNAs and target genes were analyzed using the 2^−ΔΔCt^ method. Furthermore, all qPCR data were analyzed for significant differences using the independent-sample *t*-test in GraphPad (v.8.0.2); *p* < 0.05 was considered as a statistically significant difference. In addition, all miRNA primers (one RT primer and a pair of qPCR primers for each set) were designed by Vazyme (Vazyme, NanJing, China), while mRNA primers were designed by primer3plus (http://www.primer3plus.com/cgi-bin/dev/primer3plus.cgi) and PrimerSelect (Plasterer, 1997; Friedman et al., 2009).

#### Western Blot

About 150–250 embryos from 10 female mice at each stage were collected and treated with RIPA lysis buffer. The total protein was extracted, then the protein concentration was measured using the BCA method. Then, these proteins (5 µg/well) were boiled at 95°C for 10 min, separated by 10% w/v SDS-PAGE (sodium dodecyl sulfate polyacrylamide gel electrophoresis) at 110 V for 60 min and transferred to hydrophobic polyvinylidene difluoride membranes (Hybond-P) at 220 mV for 120 min. After blocking in 5% bovine serum albumin (BSA) at room temperature for 1 h, membranes were incubated with primary antibodies, anti-PTEN (abcam, ab267787) and anti-β-actin (abcam, ab8226) at 4°C overnight and incubated with secondary antibody for 1 h. Finally, the proteins were visualized by Gel DOC™ XR^+^ with Image Lab™ Software (Bio-Rad, HL, United States) and analyzed by ImageJ software ver. 1. 8.0 (National Institutes of Health, WI, United States) (Hammer et al., 2005).

#### Clinical Data and Statistical Methods

The analysis of clinical data included general demographic characteristics, sperm parameters, and clinical embryologic development outcomes. Categorical data were expressed as frequency and percentage, while differences in these variables between the fresh and frozen-thawed groups were assessed by Pearson's chi-square χ^2^ analysis, using Fisher's exact test for expected frequencies <5. Continuous data were represented as mean ± SD, and between-group differences were assessed by the *t*-test and Wilcoxon rank sum test. All clinical statistical analyses took place using SPSS (SPSS Inc., IL, United States). A two-sided *p*-value of <0.05 was taken to indicate statistical significance (Cihan and Esen, 2021).

### Result

#### Effect of Sperm Cryopreservation on Embryonic Development in Clinical Assisted Reproductive Technology Cases and Mouse IVF Embryos

In this retrospective cohort study of human sperm cryopreservation in assisted reproductive technology (ART) cases, clinical data of 999 fresh oocytes with fresh sperm and 128 fresh oocytes with donor sperm (frozen-thawed sperm) in IVF cases as well as 538 fresh oocytes with fresh sperm and 836 fresh oocytes with donor sperm in ICSI cases were analyzed. There were no significant between-group differences in the general clinical indicators (Supplementary Table S1). The motility rate after being frozen-thawed significantly decreased (Supplementary Table S2). The results for embryonic development were as follows: cleavage rate and embryo rate were similar between the two groups; fertilization rate was 68.37% with fresh sperm, and this significantly decreased to 53.91% with frozen-thawed sperm in IVF cases; meanwhile in ICSI cases, the respective figures were 79% with fresh sperm and a significant decrease to 70.69% with frozen-thawed sperm. These results reflected that spermatozoa cryopreservation in humans made a notable effect of fertilization in both IVF and ICSI cases. Moreover, the blastocyst formation rates in IVF cases were similar, but were significantly decreased in ICSI cases, from 50.72% with fresh sperm to 34.31% with frozen-thawed sperm, further indicating the effect of sperm cryopreservation on blastocyte development, especially in ICSI cases (Table 1).

**TABLE 1:** Reproductive outcomes for women in the frozen-thawed sperm group and fresh sperm group.

Variable	Fresh sperm vs. frozen-thawed sperm (IVF)			Fresh sperm vs. frozen-thawed sperm (ICSI)		
	Fresh sperm	Frozen-thawed sperm	*p*-value	Fresh sperm	Frozen-thawed sperm	*p*-value
Fertilization rate	683/999(68.37%)	69/128(53.91%)	**0.001**	425/538(79.00%)	591/836(70.69%)	**0.001**
Cleavage rate	664/683(97.22%)	68/69(98.55%)	1.000	415/425(97.65%)	584/591(98.82%)	0.152
Day3 qualified embryo rate	587/664(88.40%)	61/68(89.71%)	0.748	347/415(83.61%)	500/584(85.62%)	0.385
Day3 high qualified embryo rate	442/664(66.57%)	48/68 (70.59%)	0.502	239/415(57.59%)	347/584(59.42%)	0.563
Blastocyst formation rate	107/259 (41.31%)	13/28 (46.43%)	0.602	105/207(50.72%)	70/204(34.31%)	**0.001**

ICSI, intracytoplasmic sperm injection; IVF, *in vitro* fertilization. Bold values means P<0.05.

In mouse embryonic experiments, we analyzed Day1, Day2, and Day3.5 embryos between the frozen group (frozen-thawed sperm were co-cultured with fresh oocytes during IVF) and fresh group (fresh sperm were co-cultured with fresh oocytes during IVF). Compared to the fresh group, there were decreased odds of embryos formation after sperm cryopreservation [88.54% of Day1 embryo rate in the fresh group vs. 69.82% in the frozen group; 82.86% vs. 57.20% of Day2 embryo rate and 80.86% vs. 59.21% of Day3.5 embryo rate, respectively] (Table 2). These data of clinical ART cases and mouse experiments showed that frozen-thawed sperm significantly affected early embryonic development.

**TABLE 2:** Reproductive outcomes for mice in the frozen-thawed sperm group and fresh sperm group.

Variable	Fresh group	Frozen group	*p*-value
Day1	309/349(88.54%)	236/338(69.82%)	**0.001**
Day2	290/350(82.86%)	155/271(57.20%)	**0.001**
Day3.5	340/420(80.95%)	90/152(59.21%)	**0.001**

Bold values means *P*<0.05.

#### miRNA Expression Characteristics of Cryopreservation on Sperm Across Humans and Mice

**FIGURE 1:**
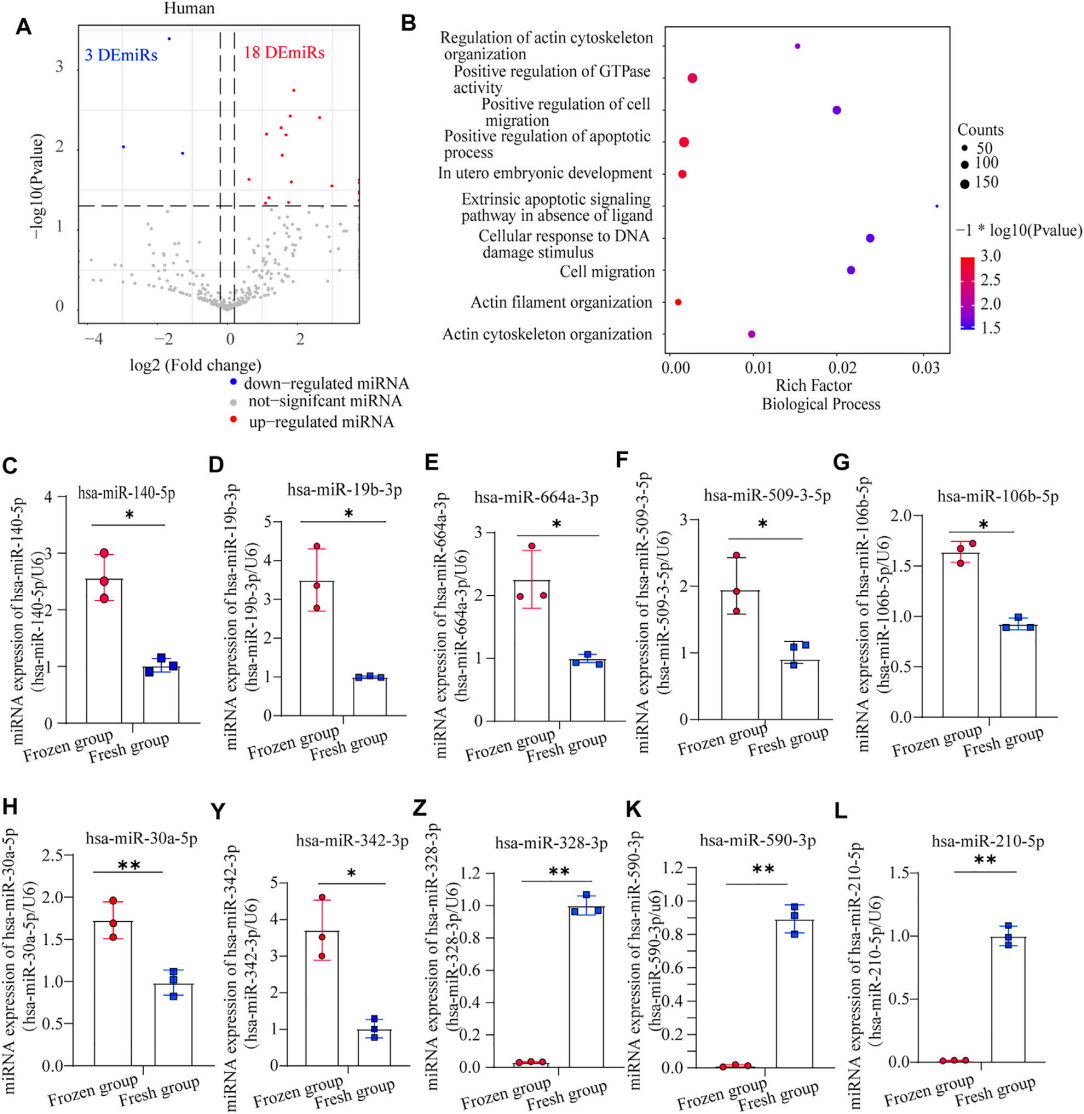
Analysis of differentially expressed miRNAs (DEmiRs) in human sperm: **(A)** volcano plot of DEmiRs between human fresh sperm and frozen-thawed sperm; **(B)** biological process (BP) enrichment analysis of DEmiRs in human sperm; **(C–L)** the expression levels of miR-140-5p, miR-19b-3p, miR-664a-3p, miR-509-3-5p, miR-106b-5p, miR-30a-5p, miR-342-3p, miR-328-3p, miR-590-3p, and miR-210-5p in human sperm, as detected by RT-PCR (mean ± SD, ∗*p* < 0.05, ∗∗*p* < 0.01).

To investigate the molecular effects of sperm cryopreservation, we examined and compared the miRNA expression of frozen-thawed and fresh sperm in humans and mice. Twenty-one DEmiRs were detected in human frozen-thawed sperm compared with fresh sperm, with 18 upregulated and 3 downregulated miRNAs (Figure 1A). qRT-PCR validated that hsa-miR-140-5p, miR-19b-3p, miR-664a-3p, miR-509-3-5p, miR-106b-5p, miR-30a-5p, and miR-342-3p were significantly upregulated, while hsa-miR-328-3p, hsa-miR-590-3p and hsa-miR-210-5p were downregulated in the frozen group (Figures 1C–L). These DEmiRs were mainly involved in the following: extrinsic apoptotic signaling pathway in the absence of ligands, cellular response to DNA damage stimulus, actin cytoskeleton organization, *in utero* embryonic development, positive regulation of cell migration, and regulation of small GTPase mediated signal transduction (Figure 1B). In addition, up-DEmiRs were closely related to *in utero* embryonic development, cellular response to DNA damage stimulus, actin filament organization, cell cycle, and positive regulation of apoptotic process; (Supplementary Figure 1A) down-DEmiRs were enriched in nervous system development, negative regulation of G0 to G1 transition, negative regulation of extrinsic apoptotic signaling pathway *via* death domain receptors, and DNA damage checkpoint, (Supplementary Figure 1B) similar to the biological process (BP) result of up-DEmiRs.

**FIGURE 2:**
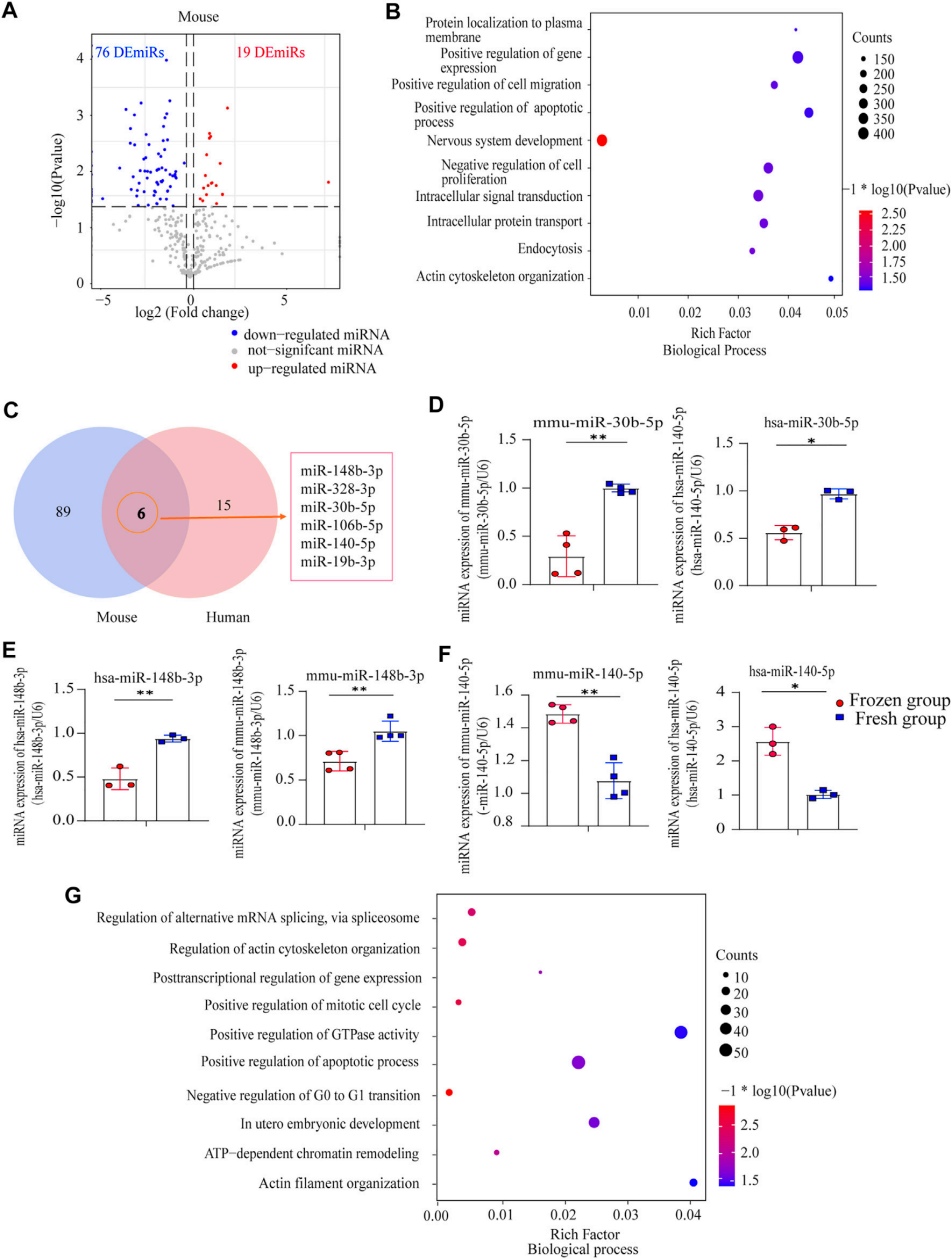
Analysis of differentially expressed miRNAs (DEmiRs) in human sperm and mouse sperm: **(A)** Volcano plot of DEmiRs between mouse fresh sperm and frozen-thawed sperm; **(B)** biological process (BP) enrichment analysis of DEmiRs in mouse sperm; **(C)** Venn analysis of human sperm and mouse sperm; **(D)** miR-30b-5p expression levels in human sperm and mouse sperm, as detected by RT-PCR (mean ± SD, ∗*p* < 0.05, ∗∗*p* < 0.01; **(E)** miR-148b-3p expression levels in human sperm and mouse sperm, as detected by RT-PCR (mean ± SD, ∗*p* < 0.05, ∗∗*p* < 0.01); **(F)** miR-140-5p expression levels in human sperm and mouse sperm, as detected by RT-PCR (mean ± SD, ∗*p* < 0.05, ∗∗*p* < 0.01); **(G)** BP enrichment analysis of common miRNA in mouse sperm and human sperm.

Meanwhile, 95 DEmiRs, including 19 upregulated and 76 downregulated miRNAs (Figure 2A), were found in mouse frozen-thawed sperm, which were enriched in: positive regulation of apoptotic process, actin cytoskeleton organization, protein localization to plasma membrane, and cell proliferation and development (Figure 2B).

Further detecting the common characteristics of cryopreservation across the species, we identified six homologous DEmiRs (miR-148b-3p, miR-328-3p, miR-30b-5p, miR-106b-5p, miR-140-5p, and miR-19b-3p) between humans and mice (Figure 2C). qRT-PCR confirmed that miR-30b-5p and miR-148b-3p were downregulated in human and mouse frozen-thawed sperm (*p* < 0.05; Figures 2D, E), while miR-140-5p was upregulated (*p* < 0.05; Figure 2F); these three common DEmiRs were enriched in the following: negative regulation of G0 to G1 transition, positive regulation of mitotic cell cycle and histone deacetylation, *in utero* embryonic development, regulation of microtubule cytoskeleton organization, and regulation of actin cytoskeleton organization (Figure 2G). These results suggested the potential influence of sperm cryopreservation on sperm miRNA profile and embryonic development across species.

#### Validation of Key miRNAs and Target Genes in Different Stages of Embryonic Development

**FIGURE 3:**
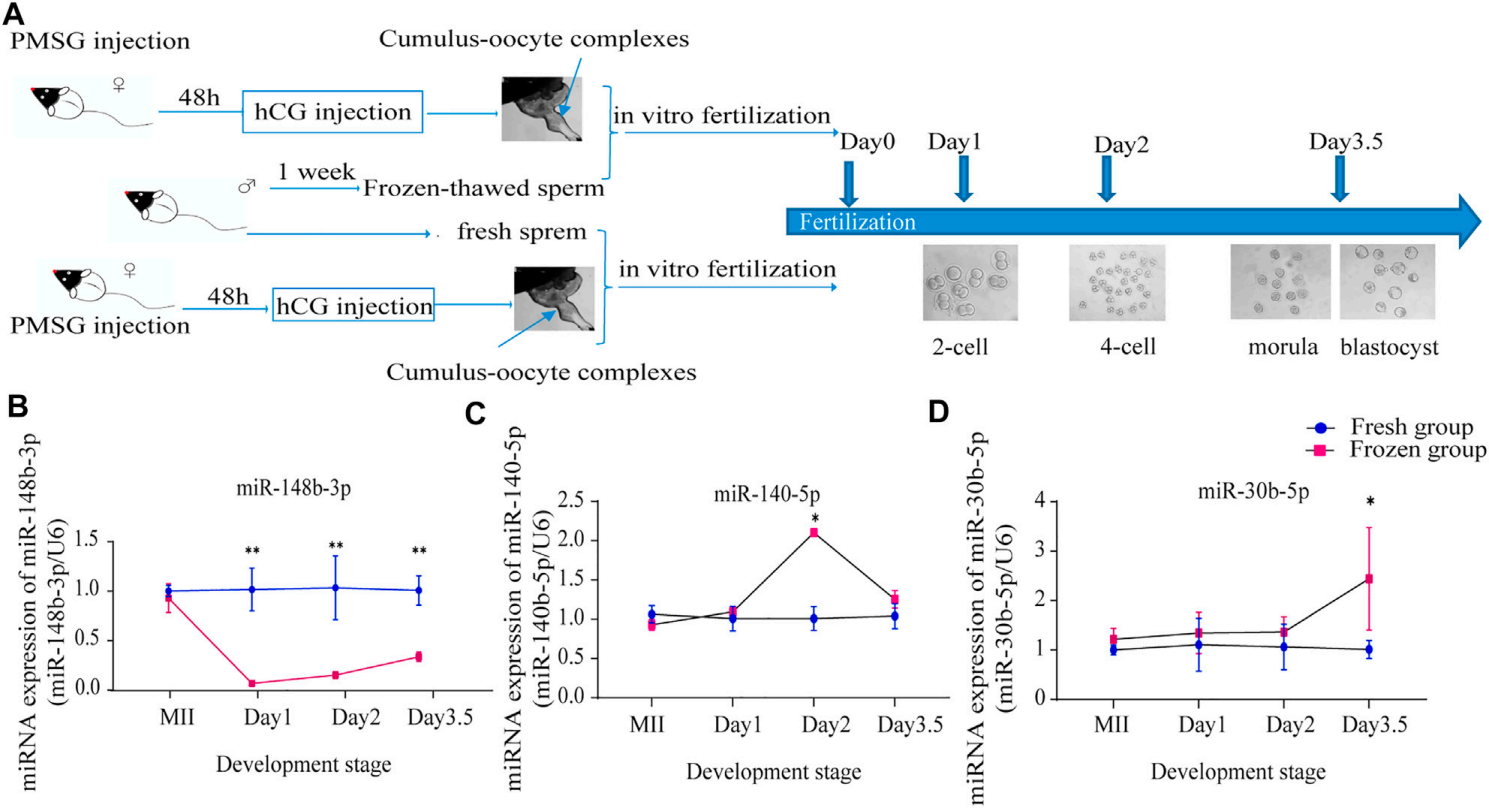
Validation of key miRNA expression levels in different embryonic developmental stages; **(A)** flow diagram of the mouse experiment; **(B–D)** miR-148b-3p **(B)**, miR-140-5p **(C)** and miR-30b-5p **(D)** expression levels in MII oocytes, Day1 embryos, Day2 embryos, and Day3. 5 embryos as detected by RT-PCR (mean ± SD, *p < 0.05, **p < 0.01).

We set out to further validate the influence of three key miRNAs related to sperm cryopreservation on mouse embryo development. Day1, Day2, and Day3.5 embryos after fertilization using fresh sperm and frozen-thawed sperm with fresh oocytes (fresh group and frozen group) were validated by qRT-PCR, respectively (Figure 3A). Notably, consistent with frozen-thawed sperm, miR-148b-3p expression levels were significantly lower starting from Day1 to Day3.5 in the frozen group compared with the fresh group (*p* < 0.01; Figure 3B); miR-140-5p expression levels were increased at Day2 (*p* < 0.05; Figure 3C), while miR-30b-5p expression levels were significantly upregulated at Day3.5 (*p* < 0.05; Figure 3D) in the frozen group. Thus, miR-148b-3p was chosen for further investigation.

**FIGURE 4:**
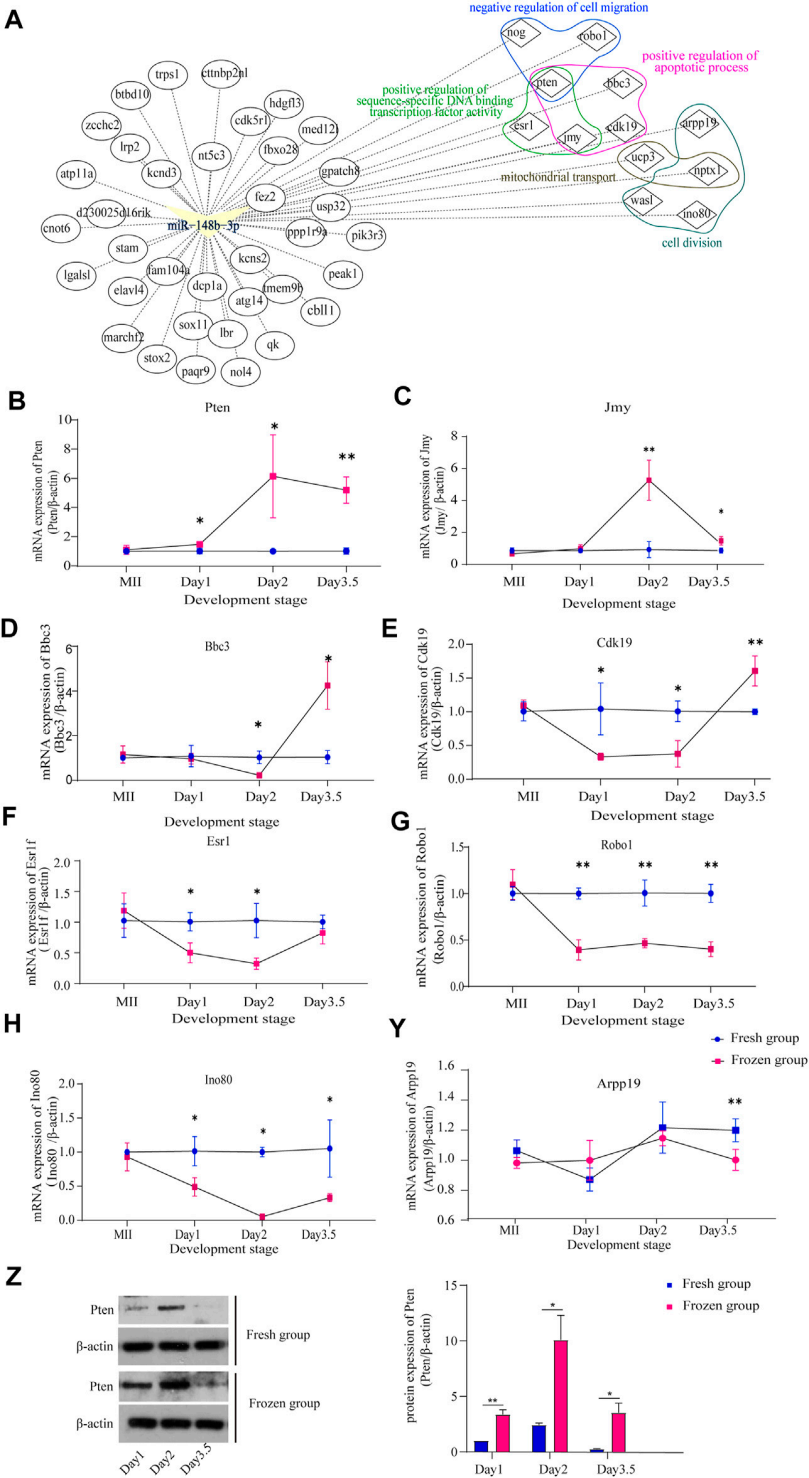
Validation of target gene expression levels in different embryonic developmental stages; **(A)** the interaction of miR-148b-3p, target genes, and pathways; **(B–Y)** the expression levels of Pten, Jmy, Bbc3, Cdk19, Esr1f, Robo1, Ino80, and Arpp19 in MII oocytes, Day1, Day2, and Day3.5 embryos after fertilization as detected by RT-PCR (mean ± SD, ∗*p* < 0.05, ∗∗*p* < 0.01). **(E)** The protein expression levels of Pten, in Day1, Day2, and Day3.5 embryos after fertilization as detected by Western blot (∗*p* < 0.05, ∗∗*p* < 0.01).

Next, the interactions between miR-148b-3p and its potential target genes as well as pathways were predicted using miRwalk3.0 and DAVID. The result showed that a total of 48 targeted genes were associated with the negative regulation of cell migration, the positive regulation of sequence-specific DNA binding transcription factor activity, mitochondrial transport, and positive regulation of apoptotic process and cell division (Figure 4A). We examined the expression level of potential genes which enriched in these significant terms. Considering that miRNA negatively regulates gene expression by degrading target mRNA (Wu et al., 2020), we found that the expression level of Pten was significantly increased at all developmental stages (Figure 4B); other genes, such as Jmy, were only upregulated on Day2, while Bbc3 and Cdk19 were downregulated firstly and upregulated on Day3.5 (Figures 4C–F). We also detected some downregulated target genes of miR-148b-3p on Day1 and Day2, including Esr1, Robo1, and Ino80, and on Day3.5 including Robo1, Ino80, and Arpp19 (Figures 4G–Y). Previous articles have proved that PTEN serves as a downstream target of miR-148b-3p in mouse and human cells. Thus, we further verified the protein level of PTEN on the early embryo using Western blot. The result showed that PTEN was increased on Day2 than on Day1 but sharply decreased in Day3.5 in both fresh and frozen-thawed groups, while PTEN was significantly increased at each stage of frozen-thawed group than in the fresh group (Figure 4Z). In summary, Pten may be a critical gene regulated by miR-148b-3p since it was enriched in the PI3K/AKT and other key pathways of early embryonic development.

#### miRNA Expression Characteristics of Cryopreserved Mouse Gametes

**FIGURE 5:**
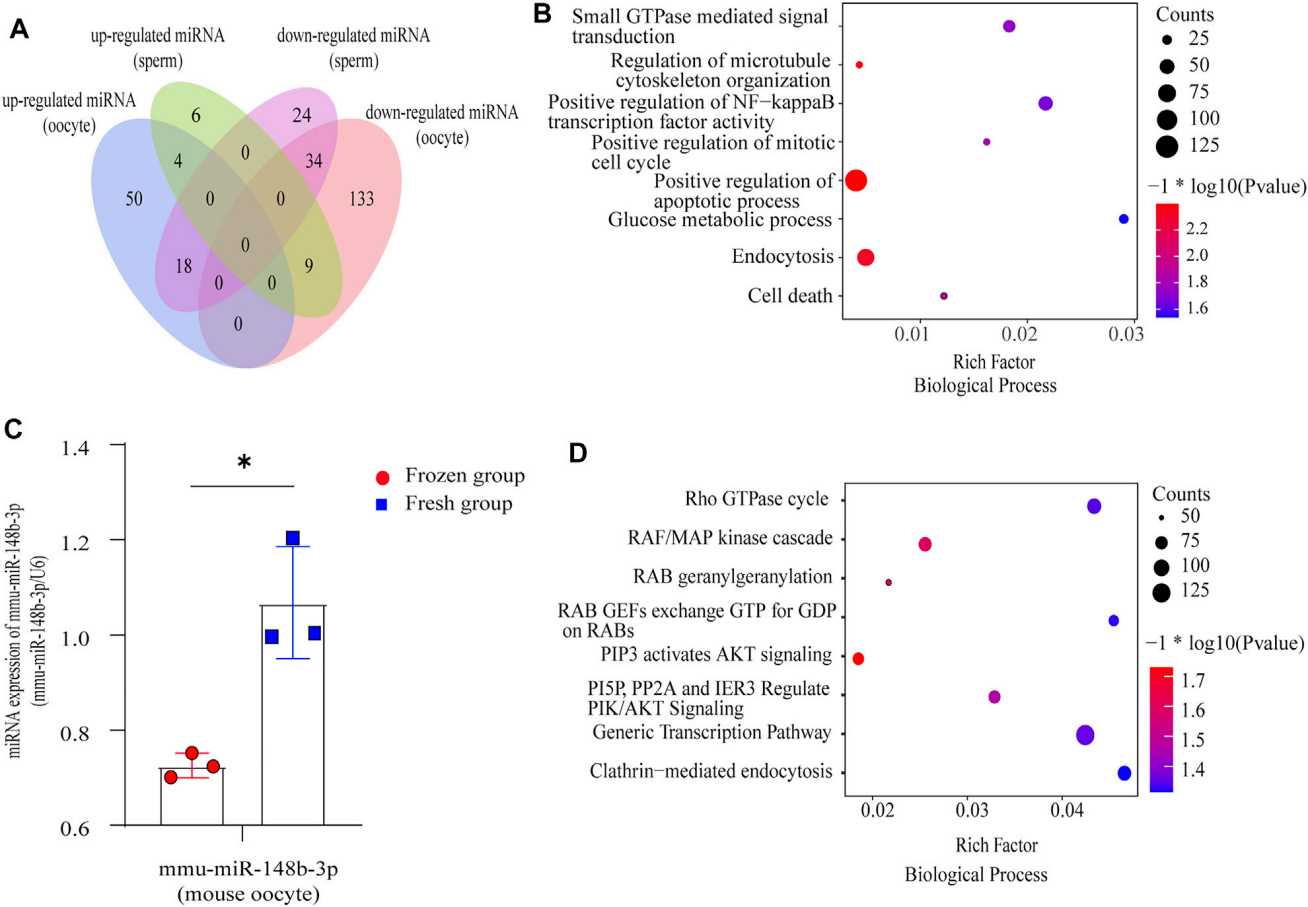
Analysis of miRNAs in mouse gametes: **(A)** Venn diagram of common DEmiRs between mouse sperm and mouse oocytes; **(B)** BP enrichment analysis of common upregulated DEmiRs in mouse gametes; **(C)** miR-148b-3p expression levels between the fresh group and frozen group in mouse oocytes, as detected by RT-PCR (mean ± SD, ∗*p* < 0.05, ∗∗*p* < 0.01). **(D)** BP enrichment analysis of common downregulated DEmiRs in mouse gametes.

To explore the common effects of cryopreservation on mouse gametes, we also performed miRNA sequencing on cryopreserved oocytes. The results identified 248 DEmiRs (72 upregulated and 176 downregulated) in frozen-thawed oocytes (Figure 5A). In addition, we combined the DEmiRs between mouse sperm and oocytes and found 4 commonly upregulated and 34 downregulated DEmiRs in frozen gametes compared with fresh gametes (Figure 5A). Functional enrichment analysis showed that these upregulated miRNAs were mainly associated with the following terms: small GTPase mediated signal transduction, regulation of microtubule cytoskeleton organization, positive regulation of NF-kappaB transcription factor activity, regulation of apoptotic process, positive regulation of mitotic cell cycle, and glucose metabolic process (Figure 5B). The downregulated miRNAs mainly affected PI5P, PP2A, and IER3 regulate PI3K/AKT signaling, PIP3 activates AKT signaling, RAB GEFs exchange GTP for GDP on RABs, Rho GTPase cycle, and RAF/MAP kinase cascade (Figure 5D). Notably, using qRT-PCR, we also validated that the expression level of miR-148b-3p was significantly downregulated in frozen-thawed oocytes (Figure 5C). The data described above hinted that the effects on miRNA expression profiles may be similar for both sperm and oocyte cryopreservation. These data in cryopreserved sperm and oocytes also indicated that downregulation of miR-148b-3p induced by cryopreservation was conserved in mouse gametes and may further affect embryonic development.

## Discussion

Fertility preservation is a meaningful option to retain fertility for people with medical needs such as the damage of chemotherapy and radiation in cancer therapy, the epidemic on reproductive health, or with social needs such as the postponement of childbearing. Sperm cryopreservation is a well-used technique in reproductive medicine (Baram et al., 2019). Increasing evidence suggests that cryopreservation greatly affects sperm as well as embryo development; however, to date, the molecular mechanisms have not been completely understood (García-López and del Mazo, 2012).

Many previous studies have found that there was no difference between frozen and fresh sperm on fertilization and embryonic development (Sakamoto et al., 2005; Yu et al., 2018), but other studies, including ours, have found that cryopreservation had an impact (Loomis et al., 1983; De Croo et al., 1998; Kanto et al., 2009; Roca et al., 2013). Our clinical data of ART cases and mouse embryonic experiments indicated that sperm cryopreservation does indeed affect sperm motility and early embryonic development, especially the fertilization rate and blastocyst formation rate, but the success rate of the frozen sperm group was still high, which is an important reason for the wide application of frozen sperm. In addition, a statistically similar blastocyst rate was shown in blastocyst formation rate between the clinical data of the two IVF groups, which may be the reason that the tremendous difference (107/259 vs. 13/28; 9-fold) in sample size induced that the statistical power (1-β) was lower than 0.8 (Song and Gilbody, 1998), leading to no significant difference results. Furthermore, as a limitation of this study, we only focused on the effect of frozen sperm on human and mouse preimplantation embryo development and did not check the final clinical outcome. In view of the strong self-correction ability, the effect of frozen sperm on early embryo development may be corrected and further reduce the difference with fresh sperm (Grau et al., 2011).

miRNAs can suppress translation in gene expression during mammalian developmental transitions, including the oocyte-to-zygote (OZA) transition, ZGA transition, and the development of embryos. Increasing evidences found that many miRNAs associated with the cryopreservation of sperm in mammals are related with fertility, the development of early embryo, and pregnancy. Among the 11 DEmiRs that we identified in human frozen-thawed sperm, hsa-miR-106b-5p (Wei et al., 2017; He et al., 2020), hsa-miR-590-3p (Shi X. et al., 2020), hsa-miR-328-3p (Bai et al., 2021), and hsa-miR-140-5p (Nie et al., 2019; Jiao et al., 2020) may cause a decreased fertilization rate in IVF and ICSI cases when using cryopreserved sperm since these miRNAs are all related to cell cycle and mitosis. miR-106b-5p is a key miRNA morula and blastocyst developmental state, having potential interactions with embryo-expressed genes (Sanchez et al., 2021). Upregulated miR-328-3p is a biomarker in teratozoospermia and may decrease fertilization and embryo formation rate (Corral-Vazquez et al., 2019). Moreover, for the three common DEmiRs between human and mouse sperm, it is reported that overexpression of miR-140-5p significantly inhibits cell proliferation and promotes cell apoptosis in chronic myeloid leukemia cells (Geng et al., 2020; Jiao et al., 2020), while miR-30b-5p can promote cell proliferation and cell cycle progression in cancer (Qin et al., 2017), and miR-148b-3p is correlated with fertility rate (Chen et al., 2018; Sekulovski et al., 2021). More importantly, in our study, downregulation of miR-148b-3p, according to qRT-PCR analysis, in both human and mouse frozen-thawed sperm may still affect the expression of miR-148b-3p during subsequent embryonic development, leading to the significantly lower embryo formation rate. Then, we screened out some target genes of miR-148b-3p, including Pten, Jmy, Bbc3, and Cdk19, which may be associated with mouse embryonic development. Available evidence shows that Cdk19 (He et al., 2020) and Bbc3 (Klimovich et al., 2020) are significantly upregulated by qRT-PCR at Day3.5. These are essential factors in early embryonic development, regulating trophoblast invasion and reflecting developmental changes in the early embryo. Pten (Qi et al., 2020) and Jmy (Lin et al., 2015), which were significantly increased from Day2 to Day3.5, are key genes regulating cell apoptosis during embryo development. In accordance with previous articles, we proposed that the target genes of downregulated miR-148b-3p should be increased in the frozen group compared to the fresh group during embryonic developmental stages (Chen et al., 2018). Moreover, previous research had proved that Pten serves as a downstream target of miR-148b-3p in mouse and human cells (Shan et al., 2021; Zhang and Mao, 2021), and our result has showed that the protein level of PTEN in the early embryo was increased in the frozen group. In all, Pten, a significantly upregulated gene in the early embryo, may be the key target gene regulated by miR-148b-3p, which may be involved in early embryonic development, cell migration, epithelial morphogenesis, and cell apoptosis in sperm cryopreservation (Di Cristofano et al., 1998). Moreover, downregulated miR-148b-3p in sperm, according to recent studies, may further enhance the role of Pten in early embryonic development and mediating the EGA and implantation of embryo by phosphatidyl-inositol-3-kinase PI3K/AKT signaling pathway (Qi et al., 2020; Zhang and Mao, 2021).

Oocyte freezing, including “social egg freezing” and “medical egg freezing,” is an important technique (Kool et al., 2018; Barberet et al., 2020). As clinical human samples were difficult to obtain, mouse oocyte miRNAs were analyzed in this study. miRNA sequencing data in mouse gametes confirmed that these common DEmiRs may similarly and negatively affect apoptosis, the PI3K/AKT signaling pathway, and cell cycle pathway in gamete integrity and later embryonic development. The key miRNA to be validated in sperm, miR-148b-3p, was also found to be downregulated in mouse frozen-thawed oocytes, so it may also affect apoptosis and the ATP pathway in embryos. In addition, considering that frozen-thawed oocytes are usually fertilized by ICSI in clinical ART cases and frozen-thawed sperm were used for IVF in this study, the gene expression of embryos with frozen-thawed oocytes were not further explored.

In conclusion, the clinical effects and the miRNA profile of sperm cryopreservation were described in the present study. miR-148b-3p was found to be downregulated in both mouse sperm and oocytes and was associated with subsequent fertilization and embryonic development. Furthermore, Pten might be a direct and functional target of miR-148b-3p during early embryonic development following fertilization by cryopreserved sperm.

## Data Availability

The miRNA sequencing data have been deposited in the NCBI Sequence Read Archive (accession number of BioProject is PRJNA755807, https://www.ncbi.nlm.nih.gov/bioproject/?term=PRJNA755807) and National Genomics Data Center (BioProject: PRJCA006219, https://ngdc.cncb.ac.cn/gsa-human/s/Wy8s55rJ).

## Supplementary Material

Supplementary Figure S1 Biological Process (BP) enrichment analysis of up-DEmiRs and down-DEmiRs in human sperm: **(A)** BP enrichment analysis of up-DEmiRs in human sperm; **(B)** BP enrichment analysis of down-DEmiRs in human sperm.

Supplementary Table S1 Characteristics of participants in sperm cryopreservation study; Table S2: semen parameters of participants in sperm cryopreservation study.
