# Supplementary material for: Effect of Sperm Cryopreservation on miRNA Expression and Early Embryonic Development
Source: Front Cell Dev Biol. 2021 Dec 22;9:749486. doi: 10.3389/fcell.2021.749486 (PMC8728010; doi:10.3389/fcell.2021.749486)
Supplement: Supplementary file 1 [file DataSheet1.docx]

Supplementary Material

# Supplementary Figure


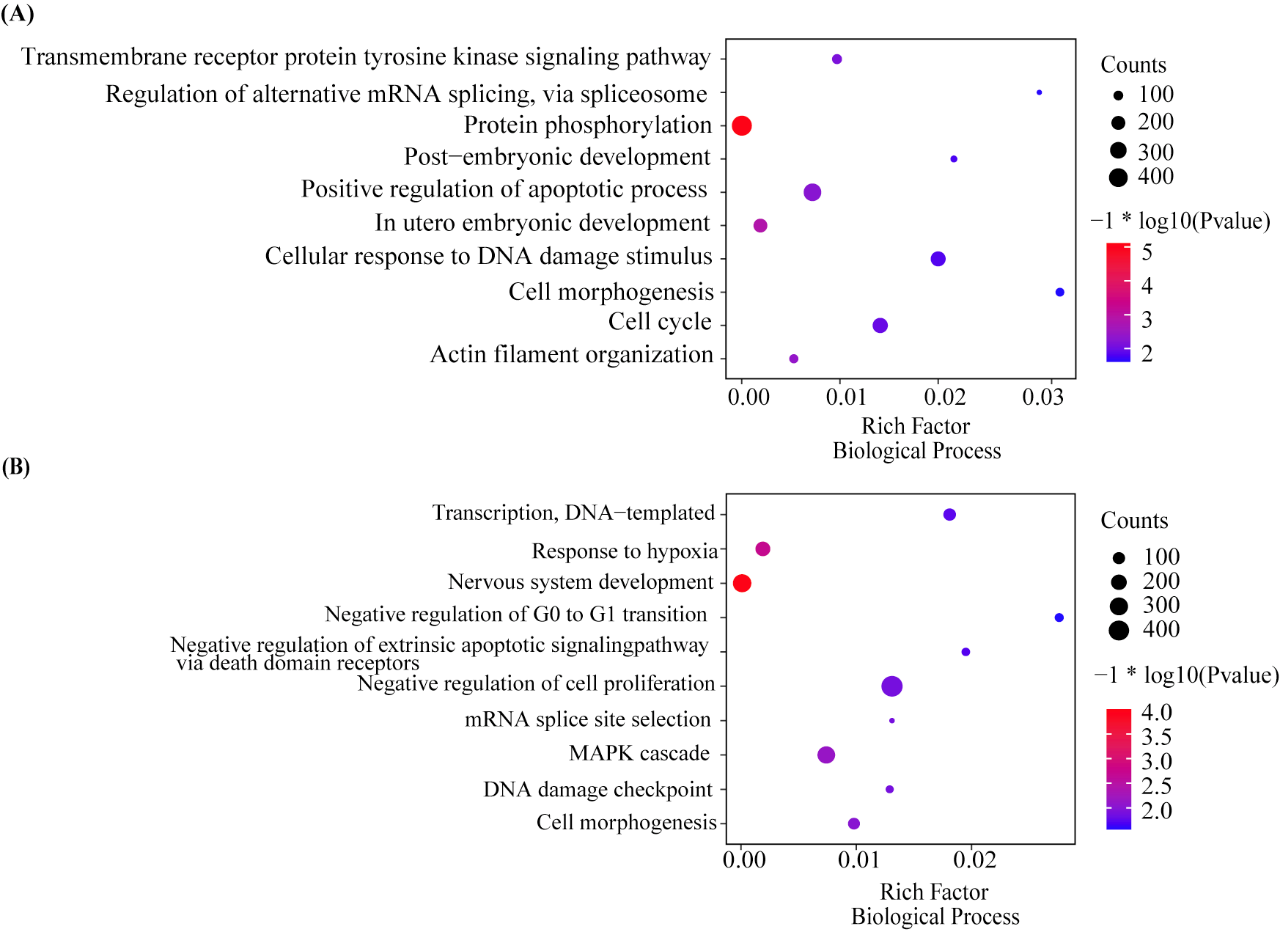


Supplementary Figure| Biological Process (BP) enrichment analysis of up-DEmiRs and down-DEmiRs in human sperm: (A) BP enrichment analysis of up-DEmiRs in human sperm; (B) BP enrichment analysis of down-DEmiRs in human sperm.

# Supplementary Tables

Supplementary Table 1| Characteristics of participants in sperm cryopreservation study

|  | Fresh sperm  (IVF) | Frozen-thawed sperm (IVF) | Fresh sperm (ICSI) | Frozen-thawed sperm (ICSI) |
| --- | --- | --- | --- | --- |
| Female characteristics | | | | |
| The NO. of female patients | 67 | 9 | 40 | 56 |
| Age at inclusion, years (mean (SD)): | 28.85 (2.87) | 27.67 (5.58) | 28.88 (3.04) | 27.75 (3.36) |
| Body mass index (mean (SD)) | 22.14 (3.31) | 22.08 (2.53) | 22.22 (3.19) | 22.07 (3.20) |
| Duration of infertility (years; mean (SD)) | 3.13 (1.97) | 3.56 (2.11) | 3.58 (2.35) | 4.16 (2.90) |
| FSH (IU/L) | 5.90 (2.63） | 6.98(3.17） | 5.81(2.53） | 5.82 (2.16） |
| LH (IU/L) | 5.21(2.98） | 4.87(1.68） | 5.23(2.37） | 4.69 (2.59) |
| E2(pmol/L） | 167.31(116.52） | 137.53 (62.41） | 167.98 (130.17） | 192.54 (164.19） |
| T(nmol/L） | 1.32 (0.65) | 1.96 (1.38) | 1.52 (0.61) | 1.41 (0.68) |
| P(nmol/L） | 1.96 (1.52) | 1.32 (0.50) | 2.15 (1.49) | 1.77 (1.29) |
| Primary cause of infertility | | | | |
| Female factor infertility-Tubal factor | 39 (58.21%) | 3 (33.33%) | 25 (62.5%) | 29 (51.79%) |
| Female factor infertility -Sequelae of pelvic inflammatory disease | 26 (38.81%) | 4 (44.44%) | 13 (32.5%) | 19 (33.93%) |
| Other | 2 (2.99%) | 2 (22.22%) | 2 (5%) | 8 (14.29%) |

FSH: Follicle-Stimulating Hormone; LH: Luteinizing Hormone; E2: Estradiol; SD: standard deviation

Supplementary Table 2| Semen parameters of participants in sperm cryopreservation study

|  | Fresh sperm VS Frozen-thawed sperm (IVF) | | | Fresh sperm VS Frozen-thawed sperm (ICSI) | | |
| --- | --- | --- | --- | --- | --- | --- |
| Variable | Fresh sperm | Frozen-thawed sperm | P.value | Fresh sperm | Frozen-thawed sperm | P.value |
| PR (Mean±SD) | 79.63±8.11 | 25.57±8.93 | <0.001 | 76.5±12.66 | 17.22±5.98 | <0.001 |
| NP (Mean±SD) | 10.35±4.46 | 18.95±6.23 | <0.001 | 11.2±5.51 | 16.85±8.18 | <0.001 |
| IM (Mean±SD) | 10.31±6.02 | 55.47±12.65 | <0.001 | 12.18±11.67 | 65.93±10.76 | <0.001 |

PR: Progressive motility; NP: Non-progressive motility; IM: Immotility.
